# The quality of reporting of randomized controlled trials of electroacupuncture for stroke

**DOI:** 10.1186/s12906-016-1497-y

**Published:** 2016-12-09

**Authors:** Jing-jing Wei, Wen-ting Yang, Su-bing Yin, Chen Wang, Yan Wang, Guo-qing Zheng

**Affiliations:** Department of Neurology, the Second Affiliated Hospital and Yuying Children’s Hospital of Wenzhou Medical University, Wenzhou, China

**Keywords:** Electroacupuncture, Stroke, Randomized controlled trial, Methodology

## Abstract

**Background:**

Electroacupuncture (EA), as an extension technique of acupuncture based on traditional acupuncture combined with modern electrotherapy, is commonly used for stroke in clinical treatment and researches. However, there is still a lack of enough evidence to recommend the routine use of EA for stroke. This study is aimed at evaluating the quality of reporting of randomized controlled trials (RCTs) on EA for stroke.

**Methods:**

RCTs on EA for stroke were evaluated by using CONSORT guidelines and STRICTA guidelines. Microsoft Excel 2010 and the R software were used for descriptive statistics analyses.

**Results:**

Seventy studies involving 5468 stroke patients were identified. The CONSORT scores ranged from 16.2 to 67.6% and STRICTA scores from 29.4 to 82.4%. The central items in CONSORT as eligibility criterion, sample size calculation, primary outcome, method of randomization sequence generation, allocation concealment, implementation of randomization, description of blinding, and detailed statistical methods were reported in 100, 6, 68, 37, 14, 10, 16, and 97% of trials, respectively. The reporting of items in STRICTA as acupuncture rationale was 1a (91%), 1b (86%) and 1c 0%; needling details 2a (33%), 2b (97%), 2c (29%), 2d (64%), 2e (100%), 2f (55%) and 2 g (66%); treatment regimen 3a (69%) and 3b (100%); other components of treatment 4a (86%) and 4b (13%); practitioner background item 5 (16%); control intervention(s) 6a (93%) and 6b (10%).

**Conclusions:**

The quality of reporting of RCTs on EA for stroke was generally moderate. The reporting quality needs further improvement.

## Background

Stroke is a major cause of death and disability in both developed and developing countries worldwide. Thrombolysis with intravenous recombinant tissue-type plasminogen activator therapy remains the only proven effective pharmacological treatment for selected acute ischemic stroke patients within a relatively short therapeutic time window of 3 to 4.5 h after the onset of stroke symptoms [1]. Furthermore, the major risk of intravenous thrombolysis treatment also remains the symptomatic intracranial hemorrhage, which is a devastating complication with high mortality. What’s more, the enormous morbidities of ischemic stroke result from the interplay between the resulting neurological impairment, the emotional and social consequences of that impairment, and the high risk for recurrence [2]. Owing to the significant health risk of stroke and the limitations of currently available conventional therapies, unprecedented attention has been attached to complementary and alternative medicine (CAM) worldwide due to its potential efficacy on stroke.

Acupuncture is one of the most commonly used CAM therapies for stroke around the world. Up to now, at least 24 systematic reviews have been published, the available evidence suggests that acupuncture is effective for improving some aspects of poststroke neurological impairment and dysfunction, although there was insufficient evidence for stroke in preventing poststroke death [3]. Especially, electroacupuncture (EA) is an extension technique of acupuncture based on traditional acupuncture combined with modern electrotherapy [4]. There are many advantages of EA such as the readily quantifiable parameters for stimulation as frequency, intensity and duration, and the therapeutic benefit of EA is commonly identified to be equivalent to manual acupuncture [5]. In some situations, EA has been shown to be more effective than manual acupuncture, particularly when strong, continued stimulation was required, as when treating stroke [6]. Thus, EA is commonly used in current clinic and research. A systematic review from our team has indicated that the available evidence potentially supported the use of EA for acute ischemic stroke [4]. However, there is still a lack of enough evidence to recommend the routine use of EA for stroke.

Both systematic reviews of high-quality randomized controlled trials (RCTs) and RCT itself, especially those with double-blind placebo controls, are commonly regarded the highest level of evidence in judging the treatment efficacy and safety of interventions. The credibility of the evidence in support of a treatment approach depends on the quality of RCTs. However, a large body of evidence indicated that the quality of reporting of RCTs remains sub-optimal [7]. Researchers have accumulated and suggested that the RCTs which were of poor methodological quality tend to exaggerate the treatment effects and result in misleading in health care at all levels [8]. So far, two studies have already been conducted to evaluate the quality of reporting of RCTs on acupuncture for stroke. In 2006, one study [9] demonstrated that the quality of reporting of 74 RCTs on acupuncture for acute stroke was generally poor. In 2014, another study [10] indicated that the quality of reporting of only 15 RCTs on acupuncture for subacute and chronic stroke was improved but some central items were still insufficiently or inadequately reported in most of the studies. However, no study has yet been conducted to assess the quality of reporting RCTs on EA for stroke. Thus, this study aimed at evaluating the quality of reporting of RCTs on EA for stroke according to the consolidated standards of reporting trials (CONSORT) statement [11] and the standards for reporting interventions in clinical trials of acupuncture (STRICTA) statement [12].

## Methods

### Information sources and search

Eight English and Chinese databases were electronically searched from their inceptions to June 2014. They are Cochrane Controlled Trials Register, PubMed, EMBASE, AMED, China National Knowledge Infrastructure(CNKI), VIP Journals Database, Wanfang Database and Chinese Biomedical Database(CBM). The search terms were listed as follows: “electroacupuncture AND (stroke OR apoplexy OR cerebrovascular accident OR cerebrovascular attack OR cerebral infarction OR intracerebral hemorrhage OR cerebral vascular disease)”. Chinese databases were also searched using the above corresponding search terms in Chinese.

### Eligibility criteria

All RCTs on EA as monotherapy or adjunct therapy for stroke compared with at least one control group as no treatment, sham/placebo EA or conventional treatment, regardless of publication status or language, were selected. The diagnostic criteria of stroke were clinically in accordance with the World Health Organization definition [13]. The diagnosis of stroke was confirmed by CT and/or MRI.

### Exclusion criteria

Studies concerning EA therapy for paresthesia, post-strokedepression, bulbar paralysis and other non-functional dysfunction were excluded. Additional exclusion criteria were animal experiment, case report, review, single-arm study, retrospective study and historical control study, duplicated publications, and quasi-randomized trial. Crossover and cluster RCTs were excluded because of the employment of the CONSORT guidelines for parallel RCTs. Searches were limited to English and Chinese publications.

### Data extraction

Two investigators underwent training in studying every item and multiple subitems listed in CONSORT2010 and STRICTA2010 to ensure the proper understanding of each standard. Each report was reviewed by two independent investigators. They extracted information according to CONSORT2010 and STRICTA2010 checklists. “1”or “0” was scored by the two authors independently to represent whether the RCT had reported the relevant item/subitem or not. “0” indicates no description of the corresponding item/subitem and “1” indicates that the author had mentioned the description of the item/subitem in the report. Investigators resolved discrepancies by consensus or consultations during the data-extraction process.

### Data analyses

Microsoft Excel 2010 and R software (Version 3.1.1) were used for descriptive statistics analyses. The overall number of RCTs which corresponded with each item was counted. Subsequently results were represented as the percentage, and 95% confidence interval (CI) of each overall rate was calculated. We also classified the included studies into two groups according to which language they were published in Chinese or English. Proportions of reported items in two groups were compared using independent sample Student’s *t*-test. Statistical calculations were performed by using SPSS (version 17.0). Level of significance was set at *P* < 0.05.

## Results

### Study selection

**Fig. 1:**
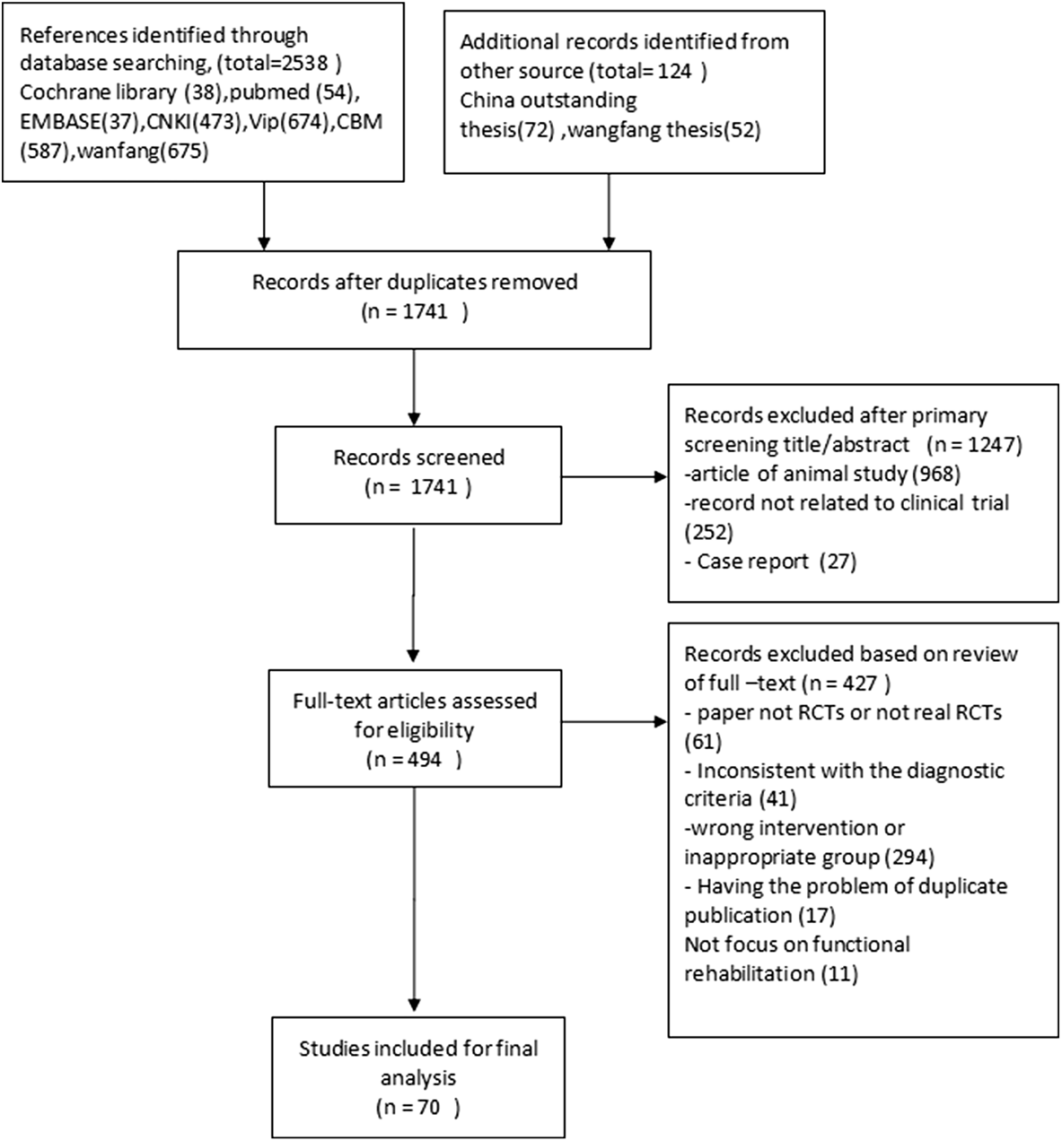
Flow diagram for the selection of articles for inclusion in the study

A total of 2662 potentially relevant articles were identified. By reviewing titles and abstracts, 2168 papers were excluded for at least one of following reasons: (1) duplicate publication, (2) animal study, (3) not clinical trial, and (4) case report. After examining the remaining 494 literatures through reading the full text, we removed 424 papers. Of which, 61 were non-randomized controlled trials, 11 were not focusing on functional rehabilitation or with other indicators, 41 were about other diseases or using an ambiguous diagnostic criteria, 17 duplicate publications, and 294 studies with other reasons. Eventually, 70 eligible RCT studies [14–83] were selected for the final analysis (Fig. 1).

### Study characteristics

Seventy studies (one article was designed with 2 comparisons) involving 5468 stroke patients were identified. For the 5468 patients, there were 2420 male and 1739 female, and with the ages ranging from 24 to 89 years old. However, the gender and age of the remaining 1309 participants could not be obtained from the primary data. Sample sizes ranged from 6 to 160 participants. In 40 studies EA was used for cerebral infarction, while in the other 30 studies EA was used for both cerebral infarction and intracerebral hemorrhage (ICH). Seventeen studies were published in English, and the other 53 studies were published in Chinese. Six studies were online Master’s thesis and not formally published [17, 40, 64, 65, 81, 82]. For the control group, WCTs were used in 62 studies and sham EA plus WCTs in 8 studies. The duration of treatment varied from 10 days to 12 weeks. Six studies conducted follow-up assessment with duration from 6 weeks to 12 months. Four studies conducted sample size calculation [20, 23, 34, 63]. Twenty-one studies reported adverse effects. In 11 studies, the discripion of the professional acupuncturists who participated in the studies was very simple and without detailed background. Nine studies reported informed consent from patients [20, 23, 24, 27, 33, 34, 64, 65, 71]. Only 6 study [27, 50, 62, 64, 65, 74] reported ethical approval. Key data are summarized in Table 1.

**Table 1:** The characteristics of the included 70 studies

Included Trials	Publicati-on language	Type of stroke	Study designs	Sample size calculation	No. of Participants (male/female); age(y)		Course of disease	Interventions(n)Drug/dosage		Course of treatment
					Trial	Control		Trial	Control	
Cao 2012 [14]	Chinese	infarction	RCT	No	40(22/18); 57.5 ± 9.8	40(24/16); 57.2 ± 9.5	<3d	electro-scalp-body-Ac	WCTs* (general supportive care, antiplatelet agents, neuroprotective agents, treatment of acute complications)	4w
Chen 2001 [15]	English	infarction	RCT	No	21(14/7); Mean 64.8	16(10/6); Mean 66.1	<3d	electro-scalp-body-Ac + WCTs#	WCTs*(specialized care)	4w
Chen 2010 [16]	Chinese	infarction	RCT	No	40 (27/13); Mean 54.4	38(28/10); Mean 55.4	≤7d	electro-body-Ac + WCTs#	WCTs* (general supportive care, antiplatelet agents, anticoagulants, neuroprotective agents)	4w
Chen C L (Unpublished Master’s thesis, 2008) [17]	Chinese	infarction ICH	RCT	No	32(20/12); 50–75	32(18/14); 50–75	<6 m	electro-body-Ac + WCTs#	Ac + WCTs* (general supportive care, specialized care, stroke rehabilitation)	4w
Dong 2011 [18]	Chinese	infarction ICH	RCT	No	75(45/30); Mean 67	75(48/27); Mean 65	<2w	electro-body-Ac + WCTs#	WCTs* (, stroke rehabilitation)	10d
Er 2010 [19]	Chinese	infarction ICH	RCT	No	30(16/14); Mean 54.2	30(18/12); Mean 56.1	1 m–3 m	electro-body-Ac + WCTs#	WCTs* (stroke rehabilitation)	6w
Fu 2010 [20]	Chinese	infarction	RCT	No	80(41/39); Mean 62.8	80(43/37); Mean 63.3	<1 m	electro-body-Ac + WCTs#	WCTs* (general supportive care, antiplatelet agents aspirin 0.1 g po qd, treatment of acute complications, stroke rehabilitation)	4w
Gao 2012 [21]	Chinese	infarction	RCT	No	82(45/37); Mean 62.4	78(42/36); Mean 62.7	3–74d	electro-scalp-body-Ac + WCTs#	WCTs* (antiplatelet agents aspirin 0.1 g po qd,)	4w
Gong 2008 [22]	Chinese	infarction; ICH	RCT	No	32 (15/17); Mean 52	31(16/15); Mean 51.4	Mean 36–38d	electro-body-Ac + WCTs#	WCTs*(stroke rehabilitation)	6w
Gosman-Hedstrom 1998 [23]	English	infarction	RCT	No	37(20/17); Mean 76.1	33(9/24); Mean 76.9	<7d	electro-scalp-body-Ac + WCTs#	WCTs*(stroke rehabilitation)	4w
Gosman-Hedstrom 1998 [23]	English	infarction	RCT	No	37(20/17); Mean 76.1	34(17/17); Mean 79	<7d	electro-scalp-body-Ac + WCTs#	Sham Ac + WCTs* (stroke rehabilitation)	10w
Guo 2009 [24]	Chinese	infarction	RCT	No	30(17/13); Mean 56.3	30(21/9); Mean 55.6	<7d	electro-body-Ac + WCTs#	WCTs* (antiplatelet agents aspirin 0.3 g po qd, a week later recuced to 0.1 g po qd, stroke rehabilitation)	14d
Hopwood 2008 [25]	English	Infarction; ICH	RCT	No	57(19/38); Mean 70.5	48(26/22); Mean 74.4	4–10d	electro-scalp-body-Ac	Sham Ac	4w
Hsing 2012 [26]	English	infarction	RCT	No	35; Mean 50	27; Mean 52	>18 m	electro-scalp-Ac	Sham Ac	5w
Hsieh 2007 [27]	English	infarction	RCT	No	30(12/18); Mean 68.8	33(20/13); Mean 70.7	<2w	electro-body-Ac + WCTs#	WCTs* (stroke rehabilitation)	4w
Hu 1993 [28]	English	infarction	RCT	No	15(15/0); 63.6 ± 6.7	15(13/2); 62.8 ± 8.0	<36 h	electro-scalp-body-Ac + WCTs#	WCTs* (general supportive care, stroke rehabilitation)	4w
Huang 2008 [29]	Chinese	infarction	RCT	No	40(21/19); Mean 63.6	40(20/20); Mean 59.9	14–90d	electro-body-Ac + WCTs#	WCTs* + ENS	4w
Huang 2011 [30]	Chinese	infarction ICH	RCT	No	35(22/13); Mean 63.2	35(19/16); Mean 65.3	Mean 7.3–8.1d	electro-scalp-body-Ac	Ac	6w
Huang 2012 [31]	Chinese	infarction	RCT	No	32(12/20) 66.59 ± 10.482;	26(16/10) 68.92 ± 10.53	<6d	electro-body-Ac + WCTs#	WCTs* (general supportive care)	4w
Jahansson 1993 [32]	English	infarction	RCT	No	38; Mean 76	40; Mean 75	<10d	electro-body-Ac + WCTs#	WCTs* (stroke rehabilitation)	10w
Jahansson 2001 [33]	English	infarction	RCT	Yes	48(29/19); Mean 76	51(25/26); Mean 76	<10d	electro-scalp-body-Ac + WCTs#	sham Ac + WCTs* (antiplatelet agents, anticoagulants, stroke rehabilitation)	10w
Jin 1999 [34]	Chinese	infarction	RCT	No	60; Mean 68	60; Mean 68	<1 m	electro-scalp-body-Ac + WCTs#	WCTs* (specialized care)	6w
Jiu 2008 [35]	Chinese	infarction ICH	RCT	No	40(23/17); Mean 62.7	40(22/18); Mean 63	<2w	Electro-body-Ac + WCTs#	WCTs* (stroke rehabilitation)	2 m
Lei2013 [36]	Chinese	infarction ICH	RCT	No	40(19/21); 48-61	40(25/15); 43–64	4–31 m	electro-body-Ac + WCTs#	WCTs* (general supportive care, stroke rehabilitation)	4w
Li 2006 [37]	Chinese	infarction	RCT	No	52(34/18); 66.8 ± 4.7	50(35/15); 67.1 ± 3.9	<1 m	electro-scalp-body-Ac + WCTs#	WCTs* (general supportive care)	3w
Li 2011 [38]	Chinese	infarction	RCT	No	30(14/16) Mean 54.4	30(13/17) Mean 55.4	<1 m	electro-body-Ac + WCTs#	WCTs* (treatment of acute complications, stroke rehabilitation)	4w
Li X Z (unpublished Master’s thesis, 2005) [39]	Chinese	infarction	RCT	No	35(18/17); Mean 61.5	35(20/15); Mean 59.7	<3d	electro-scalp-Ac + WCTs#	WCTs* (general supportive care, anticoagulantslow molecular heparin, treatment of acute complications)	10d
Liu 2007 [40]	Chinese	infarction ICH	RCT	No	38(25/13); Mean 59.4	37(14/23); Mean 56.4	<2w	Electro-body-Ac + WCTs#	WCTs* (specialized care, stroke rehabilitation)	3w
Liu 2010 [41]	Chinese	infarction ICH	RCT	No	50(32/18); Mean 61	50(35/15); Mean 63	2d–6 m	electro-scalp-Ac + WCTs#	WCTs* (stroke rehabilitation)	1 m
Long 2004 [42]	Chinese	infarction ICH	RCT	No	43(30/13); Mean 60	41(27/14); Mean 62	<7d	electro-scalp-body-Ac + WCTs#	WCTs*	7w
Luo 2012 [43]	Chinese	infarction ICH	RCT	No	10(5/5); Mean 60.5	9(5/4); Mean 62.3	2w–1 m	electro-body-Ac + WCTs#	WCTs* (stroke rehabilitation)	6w
Lv 2003 [44]	Chinese	infarction	RCT	No	29; 52–79	26; 52–79	<5d	electro-scalp-body-Ac + WCTs#	WCTs* (general supportive care, volume expansion and vasodilators, neuroprotective agents, treatment of acute complications)	1 m
Naeser 1992 [45]	English	infarction	RCT	No	10	6	1–3 m	electro-scalp-body-Ac	Sham Ac	4w
Pei2001 [46]	English	infarction	RCT	No	43(28/15); Mean 71.6	43(24/19); Mean 69.3	<7d	electro-scalp-body-Ac + WCTs#	WCTs*	4w
Peng 2007 [47]	Chinese	infarction	RCT	No	40; Mean 54	40; Mean 54	≤7d	electro-body-Ac + WCTs#	WCTs* (general supportive care, stroke rehabilitation)	12w
Peng 2009 [48]	Chinese	infarction ICH	RCT	No	30; 18–70	30; 18–70	Mean 2–3 m	electro-scalp-body-Ac	Ac	45d
Qi 2012 [49]	Chinese	Cerebral vascular disease	RCT	No	39(20/19); 60.12 ± 6.34	39(19/20); 60.23 ± 6.45	<12 m	electro–du-meridian-Ac	manual-body-Ac	20d
Sallstrom 1996 [50]	English	infarction ICH	RCT	No	26; Median 57	23; Median 58	15–71d	electro-scalp-body-Ac + WCTs#	WCTs* (stroke rehabilitation)	6w
Sang 2011 [51]	Chinese	infarction	RCT	No	40; 38–75	40; 38–75	<7d	electro-body-Ac + WCTs#	WCTs* (neuroprotective agents cerebrolysin vial 30 ml ivgtt qd, treatment of acute complications)	14d
Schaechter2007 [52]	English	infarction	RCT	No	4(3/1); 28–80	4	1–10.2y	electro-scalp-body-Ac	Sham Ac	10w
Schuler 2005 [53]	English	infarction	RCT	No	41; Mean 77.5	40; Mean 78.7	3–35d	scalp-body-Ac	Sham Ac	4w
Si 1998 [54]	English	infarction	RCT	No	20(15/5); 68 ± 10	22(18/4); 67 ± 8	<7d	electro-scalp-body-Ac + WCTs#	WCTs* (specialized care)	7d
Su 2002 [55]	Chinese	infarction ICH	RCT	No	43(27/16); 58 ± 4	40(23/17); 57 ± 5	<12 m	electro-body-Ac + WCTs#	WCTs* (general supportive care, stroke rehabilitation)	20-30d
Sun 2005 [56]	Chinese	infarction	RCT	No	40(27/13)	43(29/14)	<12 h	electro-scalp-Ac + WCTs#	WCTs* (specialized care)	12d
Sun 2012 [57]	Chinese	infarction	RCT	No	35(23/12); Mean 57.5	35(17/18); Mean 56	<3d	electro-scalp-body-Ac + WCTs#	WCTs* (specialized care)	14d
Wang 1998 [58]	Chinese	infarction	RCT	No	80; Mean 68	80; Mean 68	Mean 24d	electro-scalp-body-Ac + WCTs#	WCTs*	20d
Wang 2001 [59]	Chinese	infarction ICH	RCT	No	106; 35–80	54; 35–80;	<1y	Electro-body-Ac	Ac	6w
Wang 2003 [60]	Chinese	infarction ICH	RCT	No	32; 46–77	32; 46–77	<14d	electro-body-Ac + WCTs#	WCTs*	20d
Wang 2008 [61]	Chinese	ICH	RCT	No	45(30/15); Mean 62	45(29/16); Mean 63	<7d	electro-scalp-body-Ac + WCTs#	WCTs* (general supportive care, specialized care)	4w
Wang 2009 [62]	Chinese	infarction	Quasi-RCT	No	65(33/32); Mean 72.2	50(26/24); Mean 70.1	≤3d	electro-body-Ac + WCTs#	ENS + WCTs* (antiplatelet agents aspirin 0.1 g po qd, stroke rehabilitation)	4w
Wang Q (unpublished Master’s thesis, 2009) [63]	Chinese	infarction	RCT	Yes	24(15/9); Mean 62.4	22(14/8) Mean 57.1	<2w	electro-body-Ac + WCTs#	WCTs*(general supportive care)	4w
Wang X W (unpublished Master’s thesis, 2011) [64]	Chinese	infarction	RCT	No	31(17/14) Mean 57.4	30(19/11) Mean 60.3	<3d	electro-body-Ac + WCTs#	WCTs* (general supportive care, antiplatelet agents aspirin 0.1 g po qd, treatment of acute complications)	14d
Wayne 2005 [65]	English	infarction ICH	RCT	No	16(12/4); 38–89	17(12/5); 42–69	>6 m	electro-scalp-body-Ac	Sham Ac	10w
Wei 2008 [66]	Chinese	infarction ICH	RCT	No	46(29/17); Mean 59.4	44(23/21); Mean 56.4	2–7d	electro-body-Ac + WCTs#	WCTs* (general supportive care, specialized care, stroke rehabilitation)	5w
Wong 1999 [67]	English	infarction ICH	RCT	No	59(38/21); Mean 60.4	59(42/17); Mean 60.6	<14d	electro-body-Ac + WCTs#	WCTs*	2w
Wu 2008 [68]	Chinese	infarction ICH	RCT	No	30; 46–75	30; 46–75	>1 m	electro-body-Ac + WCTs#	WCTs* (stroke rehabilitation)	30d
Wu 2009 [69]	Chinese	infarction	RCT	No	29(16/13); Mean 56.7	29(17/12); Mean 58.5	<14d	electro-body-Ac + WCTs#	WCTs* (general supportive care, specialized care, antiplatelet agents aspirin 0.1 g po qd)	14d
Wu 2011 [70]	Chinese	infarction ICH	RCT	No	30(18/12)	30(19/11)	>3w	electro-body-Ac + WCTs#	WCTs* (general supportive care, specialized care, stroke rehabilitation)	6w
WuXL 2008 [71]	Chinese	infarction	RCT	No	32(20/12); Mean 67.2	29(19/10); Mean 66.6	<7d	electro-scalp-body-Ac + WCTs#	WCTs* + Ac	3 m
Xue 2007 [72]	Chinese	infarction; ICH	RCT	No	18(14/4); Mean 66.1	18(15/3); Mean 64.2	<2w	electro-body-Ac + WCTs#	WCTs* (stroke rehabilitation)	4w
Yu 2005 [73]	Chinese	infarction	RCT	No	16(10/6); 40–76	14(8/6); 40–75	<3d	electro-scalp-body-Ac + WCTs#	WCTs* (vasodilators, neuroprotective agents)	2w
Yue 2012 [74]	Chinese	infarction ICH	RCT	No	33(21/12); Mean 70.4	31(18/13); Mean 69.8	80-163d	electro-body-Ac	Ac	1 m
Zhang 1995 [75]	Chinese	infarction	RCT	No	40(23/17); Mean 65.8	40(22/18); Mean 68.7	<7d	electro-scalp-Ac + WCTs#	WCTs* (specialized care)	20d
Zhang 2006 [76]	Chinese	infarction ICH	RCT	No	32(17/15); Mean 62.7	25(15/10); Mean 64.5	<6 m	electro-body-Ac + WCTs#	WCTs* + Ac	30d
Zhang 2008 [77]	Chinese	infarction ICH	RCT	No	49(26/23) Mean 51.5	49(24/25) Mean 54.7	<2w	Electro-body-Ac + WCTs#	WCTs* (general supportive care, specialized care, stroke rehabilitation)	2 m
Zhang 2009 [78]	Chinese	infarction ICH	RCT	No	30(12/18); Mean 55.7	30(15/15); Mean 58.4	<3y	Electro-body-Ac + WCTs#	WCTs*(stroke rehabilitation)	1 m
Zhang 2013 [79]	Chinese	infarction	Quasi-RCT	No	45(27/18); Mean 65.5	45(30/15); Mean 63.2	<3d	electro-scalp-body-Ac + WCTs#	WCTs* (general supportive care, specialized care, neuroprotective agents)	4w
Zhang SS (unpublished Master’s thesis, 2009) [80]	Chinese	infarction	RCT	No	29(17/12); Mean 62.9	29(16/13); Mean 63.6	<10d	electro-body-Ac + WCTs#	WCTs* (general supportive care, treatment of acute complications, stroke rehabilitation)	3w
Zhang X, (unpublished Master’s thesis, 2008) [81]	Chinese	infarction	RCT	No	60(33/27); 40–80	30(17/13); 40–80	<2w	electro-body-Ac + WCTs#	WCTs* (general supportive care, antiplatelet agents aspirin 0.1 g po qd, treatment of acute complications, stroke rehabilitation)	2w
Zhao 2005 [82]	Chinese	infarction ICH	RCT	No	60(36/24); Mean 63.0	60(31/29); Mean 67.4	<2w	electro-scalp-body-Ac + WCTs#	WCTs* (general supportive care, specialized care, (treatment of acute complications, stroke rehabilitation)	1 m
Zhu 2012 [83]	Chinese	infarction ICH	RCT	No	40; 32–69	40; 32–69	<2w	Electro-body-Ac + WCTs#	WCTs* (general supportive care, specialized care)	1 m

*Ac* acupuncture, *d*day, *ICH* Intracerebral Hemorrhage, *m* month, *RCT* randomizedcontrolledtrial, *SA* scalp acupuncture, *w* week, *WCTs* western conventional treatments, *y* year. #: the same as the control group; WCT* refer to the combination of needed therapies of the following aspects: (1) General supportive care mainly include: A. airway, ventilatory support and supplemental oxygen, B. cardiac monitoring and treatment, C. temperature, D. blood pressure, E. blood sugar and F. nutrition; (2) Specialized care mainly include a variety of measures to improve cerebral blood circulation (such as antiplatelet agents, anticoagulants, fibrinogen-depleting agents, volume expansion and vasodilators, except thrombolytic agents) and neuroprotective agents; (3) Treatment of acute complications mainly include: A. brain edema and elevated intracranial pressure, B. seizures, C. dysphagia, D. pneumonia, E.voiding dysfunction and urinary tract infections and F. deep vein thrombosis.(4) Stroke rehabilitation

### Items reported according to CONSORT statement

The items reported from the 70 RCTs according to CONSORT statement are summarized in Table 2.

**Table 2:** The reporting number and percentage for each item of the CONSORT checklist of the included 70 studies

Section/Topic	Item No	Checklist item	*n*	% (n /70)	95%CI
Title and abstract	1a	Identification as a randomized trial in the title	12	17	[9 to 28]
	1b	Structured summary of trial design, methods, results, and conclusions (for specific guidance see CONSORT for abstracts)	54	77	[66 to 86]
Introduction					
Background and objectives	2a	Scientific background and explanation of rationale	63	90	[80 to 96]
	2b	Specific objectives or hypotheses	65	93	[84 to 98]
Methods					
Trial design	3a	Description of trial design (such as parallel, factorial) including allocation ratio	58	83	[72 to 91]
	3b	Important changes to methods after trial commencement (such as eligibility criteria), with reasons	0	0	[0 to 5]
Participants	4a	Eligibility criteria for participants	70	100	[95 to 100]
	4b	Settings and locations where the data were collected	58	83	[72 to 91]
Interventions	5	The interventions for each group with sufficient details to allow replication, including how and when they were actually administered	70	100	[95 to 100]
Outcomes	6a	Completely defined pre-specified primary and secondary outcome measures, including how and when they were assessed	68	97	[90 to 100]
	6b	Any changes to trial outcomes after the trial commenced, with reasons	1	1	[0 to 8]
Sample size	7a	How sample size was determined	4	6	[2 to 14]
	7b	When applicable, explanation of any interim analyses and stopping guidelines	7	10	[4 to 20]
Randomisation					
Sequence generation	8a	Method used to generate the random allocation sequence	26	37	[26 to 50]
	8b	Type of randomization; details of any restriction (such as blocking and block size)	20	29	[18 to 41]
Allocation concealment mechanism	9	Mechanism used to implement the random allocation sequence (such as sequentially numbered containers), describing any steps taken to conceal the sequence until interventions were assigned	10	14	[7 to 25]
Implementation	10	Who generated the random allocation sequence, who enrolled participants, and who assigned participants to interventions	7	10	[4 to 20]
Blinding	11a	If done, who was blinded after assignment to interventions (for example, participants, care providers, those assessing outcomes) and how	11	16	[8 to 27]
	11b	If relevant, description of the similarity of interventions	6	9	[3 to 18]
Statistical methods	12a	Statistical methods used to compare groups for primary and secondary outcomes	68	97	[90 to 100]
	12b	Methods for additional analyses, such as subgroup analyses and adjusted analyses	0	0	[0 to 5]
Results					
Participant flow (a diagram is strongly recommended)	13a	For each group, the numbers of participants who were randomly assigned, received intended treatment, and were analysed for the primary outcome	5	7	[2 to 16]
	13b	For each group, losses and exclusions after randomization, together with reasons	15	21	[13 to 33]
Recruitment	14a	Dates defining the periods of recruitment and follow-up	44	63	[50 to 74]
	14b	Why the trial ended or was stopped	2	3	[0 to 10]
Baseline data	15	A table showing baseline demographic and clinical characteristics for each group	23	33	[22 to 45]
Baseline data	16	For each group, number of participants (denominator) included in each analysis and whether the analysis was by original assigned groups	57	81	[70 to 90]
Outcomes and estimation	17a	For each primary and secondary outcome, results for each group, and the estimated effect size and its precision (such as 95% confidence interval)	2	3	[0 to 10]
	17b	For binary outcomes, presentation of both absolute and relative effect sizes is recommended	0	0	[0 to 5]
Ancillary analyses	18	Results of any other analyses performed, including subgroup analyses and adjusted analyses, distinguishing pre-specified from exploratory	1	1	[0 to 8]
Harms	19	All important harms or unintended effects in each group (for specific guidance see CONSORT for harms)	21	30	[20 to 42]
Discussion					
Limitations	20	Trial limitations, addressing sources of potential bias, imprecision, and, if relevant, multiplicity of analyses	10	14	[7 to 25]
	21	Generalisability (external validity, applicability) of the trial findings	13	19	[10 to 30]
Interpretation	22	Interpretation consistent with results, balancing benefits and harms, and considering other relevant evidence	22	31	[21 to 44]
Other information					
Registration	23	Registration number and name of trial registry	0	0	[0 to 5]
Protocol	24	Where the full trial protocol can be accessed, if available	1	1	[0 to 8]
Funding	25	Sources of funding and other support (such as supply of drugs), role of funders	14	20	[11 to 31]
Total mean score^a^			13.0 ± 4.0		

^a^Mean ± SD

#### Title and abstract

Twelve (18%) trials can be identified as random trials after reviewing the title (1a), among which 8 were in English. Fifty-four (77%) articles had abstracts that were comprised of objective, methods, results and conclusions (1b).

#### Introduction

Of the included studies, 90% provided the detailed description of backgrounds (2a). The proportion of studies with objectives (2b) was 93%.

#### Methods

Only 2 CONSORT items were described in all the included articles. One was the eligibility criterion for participants (4a) and the other was the interventions for each group with sufficient details to allow replication, including how and when they were actually administered (5). However, the proportion on the description of the patient’s allocation ratio was 58% (3a). None of the articles (0%) described the important changes after the beginning of the trial because of the recruitment (3b). Fifty-eight reports (83%) described the settings and locations where the data were collected (4b). The proportion on the description of definition of primary/secondary outcomes was 68% (6a). Four (6%) reports mentioned the method of how to determine the sample size (7a). Items on incomplete reporting were 1% (subitem 6b) and 10% (subitem 7b).

#### Randomization

Twenty studies (29%) mentioned the type of randomization as the simple random method (8b). However, the proportion of the description on sequence generation was 37% (8a), which used computer or random number table. Ten articles (14%) described the hidden mechanism by the use of opaque envelopes aiming to implement the allocation concealment (9). The detailed implementation was given in 7 articles (10%) (10). A total of 11 articles (16%) provided the description of blinding (11a), among which one was double blind (participants and evaluators) and the others were single-blind assessment. Sixty-eight studies (97%) provided the description of detailed statistical methods (12a), but no one provided methods for additional analyses (12b).

#### Result and discussion

Nine studies (13%) described the treatment progress of participants by a diagram (13a). Fifteen (21%) of these articles mentioned the number of the losses and exclusions after randomization with explanations (13b). Forty-four studies (63%) mentioned the periods of recruitment, but only 6 studies described the follow-up duration (14a). Two articles had reported a temporal interruption of the therapy because of the drop out of participants with personal reasons. Thirty-four reports (49%) offered the description of baseline data that included underlying disease or basic demographic or clinical characteristics, among which 23 studies (33%) represented the data in the form of a table (15). Fifty-seven studies (81%) described the statistics methods, including the use of intention-to-treat analysis (16). Almost all outcomes of the included reports were presented as the ratio of efficiency or means ± SD. Two papers (3%) applied 95% CI to describe the estimated value of the effect and its precision (17a). No study reported binary outcomes (17b). One study provided a kind of secondary analyses as “error type I” in statistics (18). In discussion section, 21 papers (30%) reported the occurrence of adverse events, such as acupuncture syncope, infection of puncture site and death (19). The proportions of papers reporting limitation (20), generalisability (21) and interpretation (22) were 14, 19, and 31%, respectively.

Other information: None of the papers reported the registration (23). Only 1 report (1%) gave the relevant electronic links for the obtainment of protocol (24). The proportion of paper with reporting of funding (25) was 420%.

### Items reported according to STRICTA statement

The items reported from the 70 RCTs according to STRICTA statement are summarized in Table 3.

**Table 3:** The reporting number and percentage for each item of the STRICTA checklist of the included 70 studies

Item	Detail	Total *N* = 70			Chinese *N* = 54			English *N* = 16			Chinese vs. English (*P*-value for difference)
		*n*	%(n /70)	95% CI	*n*	%(n /54)	95% CI	*n*	%(n /16)	95% CI	
1. Acupuncturerationale (Explanations and examples)	1a) Style of acupuncture (e.g. Traditional Chinese Medicine, Japanese, Korean, Western medical, Five Element, ear acupuncture, etc.)	64	91	[82 to 97]	50	93	[82 to 98]	14	88	[62 to 98]	0.530
	1b) Reasoning for treatment provided, based on historical context, literature sources, and/or consensus methods, with references where appropriate	60	86	[75 to 93]	50	93	[82 to 98]	10	63	[35 to 85]	0.033
	1c) Extent to which treatment was varied	0	0	[0 to 5]	0	0	[0 to 7]	0	0	[0 to 21]	_
2. Details of needling (Explanations and examples)	2a) Number of needle insertions per subject per session (mean and range where relevant)	25	36	[25 to 48]	18	33	[21 to 47]	7	44	[20 to 70]	0.452
	2b) Names (or location if no standard name) of points used (uni/bilateral)	68	97	[9 to 10]	52	96	[87 to 100]	16	100	[79 to 100]	0.442
	2c) Depth of insertion, based on a specified unit of measurement, or on a particular tissue level	20	29	[18 to 41]	18	33	[21 to 47]	2	12	[2 to 38]	0.060
	2d) Response sought (e.g. de qi or muscle twitch response)	46	66	[53 to 77]	39	72	[58 to 84]	7	44	[20 to 70]	0.035
	2e) Needle stimulation (e.g. manual, electrical)	70	100	[95 to 100]	54	100	[93 to 100]	16	100	[79 to 100]	_
	2f) Needle retention time	40	57	[45 to 69]	32	59	[45 to 72]	8	50	[25 to 75]	0.518
	2g) Needle type (diameter, length, and manufacturer or material)	46	66	[53 to 77]	38	70	[56 to 82]	8	50	[25 to 75]	0.222
3. Treatmentregimen (Explanations and examples)	3a) Number of treatment sessions	48	69	[56 to 79]	38	70	[56 to 82]	10	63	[35 to 85]	0.558
	3b) Frequency and duration of treatment sessions	70	100	[95 to 100]	54	100	[93 to 100]	16	100	[79 to 100]	_
4. Othercomponents of treatment (Explanations and examples)	4a) Details of other interventions administered to the acupuncture group (e.g. moxibustion, cupping, herbs, exercises, lifestyle advice)	60	86	[75 to 93]	49	91	[80 to 97]	11	69	[41 to 89]	0.098
	4b) Setting and context of treatment, including instructions to practitioners, and information and explanations to patients	9	13	[6 to 23]	4	7	[2 to 18]	5	31	[11 to 59]	0.073
5. Practitioner background (Explanations and examples)	5) Description of participating acupuncturists (qualification or professional affiliation, years in acupuncture practice, other relevant experience)	11	16	[8 to 26]	3	6	[1 to 15]	8	50	[25 to 75]	0.004
6. Control or comparator interventions (Explanations and examples)	6a) Rationale for the control or comparator in the context of the research question, with sources that justify this choice	7	10	[4 to 20]	4	7	[2 to 18]	3	19	[4 to 46]	0.302
	6b) Precise description of the control or comparator. If sham acupuncture or any other type of acupuncture-like control is used, provide details as for Items 1 to 3 above.	65	93	[84 to 98]	52	96	[87 to 100]	13	81	[54 to 96]	0.166
Total mean score^a^		10.1 ± 1.9			10.3 ± 1.8			9.6 ± 2.1			0.235

^a^Mean ± SD

#### Acupuncture rationale

Apart from several English articles, majority of the other included articles (91%) used the style of acupuncture from Traditional Chinese Medicine (1a). Eighty-six percent of the reports provided reasons for treatment based on historical context, literature sources, citing references where appropriate, and so on (1b). None of the studies had mentioned any alteration of the treatment after the beginning of the experiments (1c).

#### Needling details

Various intervention methods were used in EA treatment group, and were mainly as follows: EA plus conventional theory, EA plus acupoint injection, scalp EA plus acupoint injection, and EA plus internal carotid injection. All the 70 included reports provided the type of needle stimulation, including electrical acupuncture or electrical acupuncture combined with manual acupuncture (2e). Ninety seven percent of articles listed the names (or location if no standard name) of acupoints used at the uni/bilateral sides (2b); however, only 33% articles mentioned the number of needles, 33% (2a). Twenty nine percent of studies mentioned the depth of needle insertion (2c). The other STRICTA items on needling details were response elicited (*de qi* or muscle twitch response), 64% (2d), needle retention time, 55% (2f) and needle type, 66% (2 g).

#### Treatment regimen

All the reports mentioned the frequency and duration of treatment sessions (3b), whereas 69% articles provided the number of treatment sessions (3a).

#### Cointerventions

One item, details of other interventions, was mentioned in more than half of the reports, 86% (4a). Nine reports (13%) described some relevant information and explanations to patients, including informed consent (4b).

#### Practitioner background

Eleven articles (16%) provided vague and unspecific description on the background of acupuncturist which included expertise, duration of training and length of clinical experience (5). In the 11 articles, four mentioned that the acupuncturists were professionals, and the others mentioned the contents as expertise or duration of specific training.

#### Control intervention(s)

A total of 93% trials reported a precise description of the control or comparator (6b). Furthermore, 8 studies used sham EA as control with providing further details of items 1 to 3 in STRICTA. Ten percent of studies provided the quoted data to elucidate the rationality of contrasting and comparing other similar experiments (6a).

### Comparison of reporting quality between Chinese and English studies

The total mean score in CONSORT items failed to achieve significant differences between English studies and Chinese studies (English vs. Chinese: 15.2 ± 4.3 vs. 12.3 ± 3.6, *p* = 0.05), Table 4. However, there is statistically significant improvement in three items published in English vs. in Chinese as follows: (1a) title (56% vs.6%, *p* = 0.01), (11a) blinding (44% vs. 7%, *p* = 0.014), (13b) losses and exclusions (56% vs. 11%, *p* = 0.004). As for the other items, they all showed no statistical significant differences, Table 4.

**Table 4:** Comparison of reporting quality between Chinese and English studies (CONSORT)

CONSORT item	Chinese *N* = 54			English *N* = 16			Chinese vs. English (*P*-value for difference)
	*n*	%(n/54)	95%CI	*n*	%(n/16)	95%CI	
Title	3	6c	[1 to 15]	9	56	[30 to 80]	0.01
Methods							
Trail design	48	89	[77 to 96]	10	63	[35 to 85]	0.061
Eligibility criteria	54	100	[93 to 100]	16	100	[79 to 100]	_
Interventions	54	100	[93 to 100]	16	100	[79 to 100]	_
Primary and secondary outcome	52	96	[87 to 100]	16	100	[79 to 100]	0.442
Sample size	3	6	[1 to 15]	1	6	[0 to 30]	0.918
Generation of random sequence	22	41	[28 to 55]	4	25	[7 to 52]	0.239
Allocation concealment	6	11	[4 to 23]	4	25	[7 to 52]	0.26
Blinding	4	7	[2 to 18]	7	44	[20 to 70]	0.014
Statistical methods	53	98	[90 to 100]	15	94	[70 to 100]	0.361
Results							
Losses and exclusions	6	11	[4 to 23]	9	56	[30 to 80]	0.004
Recruitment	38	70	[56 to 82]	6	38	[15 to 65]	0.017
Numbers analysed	47	87	[75 to 95]	10	63	[35 to 85]	0.081
Harms	14	26	[15 to 40]	7	44	[20 to 70]	0.177
Limitations	4	7	[2 to 18]	6	38	[15 to 65]	0.33
Total mean score^a^	15.2 ± 4.3			12.3 ± 3.6			0.05

^a^Mean ± SD

There are no differences in proportions of items in STRICTA comparing studies in Chinese with that in English (Chinese vs. English: 10.3 ± 1.8 vs. 9.6 ± 2.1, *p* = 0.235), Table 3. Studies in Chinese have statistically siginificant improvement in the item (1b) reasoning for treatment provided (93% vs. 63%, *p* = 0.033) and (2d) response sought (72% vs. 44%, *p* = 0.035) compared with studies in English, whereas studies in English in the item (5) practitioner background (6% vs. 50%, 0.004) showed significant improvement compared with studies in Chinese, Table 3.

## Discussion

A wealth of evidence indicated the very inadequate reporting of clinical researches. For example, information on the method of random sequence generation, primary outcome, sample size calculation, randomization stated in title, allocation concealment, and adequate blinding was reported in 34, 53, 45, 33, 25, and 18% of 616 reports indexed in PubMed in 2006, respectively [84]. Especially, in RCTs of traditional Chinese medicine that include herbal medicine, acupuncture and other no medication therapies, reporting of the key methods used for adequate randomization methods, adequate allocation concealment, adequate blinding, both adequate randomization methods and allocation concealment used, and all three used was only 12, 7, 19, 4, and 3% of 2580 reports, respectively [8]. Thus, several guidelines have been recommended to help incomplete and inaccurate reporting. The CONSORT statement [11] is an evidence-based, minimum set of recommendations for reporting randomized trials to alleviate the problems arising from inadequate reporting of RCTs. It offers a standard way for authors to prepare reports of trial findings, facilitating their complete and transparent reporting, and aiding their critical appraisal and interpretation. The 2010 version of STRICTA statement [12], an official extension to the CONSORT statement, is the standards for reporting interventions in clinical trials of acupuncture to facilitate transparency in published reports, enabling a better understanding and interpretation of results, aiding their critical appraisal, and providing detail that is necessary for replication.

In the present study, the quality of reporting of 70 RCTs on EA for stroke was generally moderate. The CONSORT scores achieved by the included studies ranged from 4.7 to 91.5% according to seven subdomains, and the STRICTA scores across six subdomains ranged from 16 to 84.5%. The central items in CONSORT of eligibility criterion, sample size calculation, primary outcome, method of randomization sequence generation, allocation concealment, implementation of randomization, description of blinding, and detailed statistical methods are reported in 100, 3, 68, 37, 14, 10, 16, and 97% of 70 reports, respectively. The reporting of detail items in STRICTA of acupuncture rationale is 1a (91%), 1b (86%) and 1c 0%; of needling details is 2a (33%), 2b (97%), 2c (29%), 2d (64%), 2e (100%), 2f (55%) and 2 g (66%); of treatment regimen is 3a (69%) and 3b (100%); of other components of treatment is 4a (86%) and 4b (13%); of practitioner background is item 5 (16%); of control intervention(s) is 6a (93%) and 6b (10%). Based on the results of present study, several key items need further improvement. First, a priori sample size calculation can reduce the risk of an underpowered (false-negative) result. However, in the present study sample size calculation was reported in only 3% of all the included trials. In fact, a survey of 215 studies published in 2005 and 2006 in six general medical journals with high impact factors revealed that only 34% of 73 studies adequately described sample size calculations [85]. If the trials were not conducted with pre-trial estimation of sample size, there will be a lack of statistical power to ensure appropriate estimation of the treatment effect [86]. Thus, we suggest that an effort should be made to increase transparency in sample size calculation. Second, successful randomisation reduces selection bias at trial entry, which depends on two hinge steps-adequate sequence generation and allocation concealment, and is the crucial component of high quality RCTs [87]. In the present study method of randomization sequence generation, allocation concealment, and implementation of randomization is reported in only 37, 14, and 10% of 70 RCTs, respectively. Inadequate or unclear allocation concealment can exaggerate clinical effects in 41 and 30%, respectively [88]. Thus, proper randomization should involve both random sequence generation and complete implementation of allocation concealment to minimize bias. Third, blinding is an essential method for preventing research outcomes from being influenced by either the placebo effect or the observer bias. Trials that were not double blinded yielded larger estimates of treatment effect than trials in which authors reported double blinding (odds ratios exaggerated, on average by 17%) [88]. In the present study, only 16% of 70 trials described blinding procedure. Thus, more attentions should be paid to this situation, especially in EA trials. Fourth, item 5 in STRICTA is practitioner background that required description of participating acupuncturists in qualification or professional affiliation, years in acupuncture practice, other relevant experience. However, practitioner background was reported only in 16% trials. Thus, practitioner qualifications should be completely reported, which could increase the certainty with regard to treatment quality and safe implementation of interventions.

Currently, the evidence from the study of manual and electrical needle stimulation in acupuncture researched by an executive board of the society for acupuncture research [5] demonstrated that fundamental gaps existed in the understanding of the mechanisms and relative effectiveness between manual and electrical acupuncture, and these two techniques are not interchangeable. In 2006, Zhang et al. [9] evaluated the reporting quality of 74 RCTs on acupuncture for acute ischemic stroke, indicating that the items in CONSORT of baseline demographic and clinical characteristics, method of random sequence generation, allocation concealment, blinding procedure, sample size calculation and intention-to-treat (ITT) analysis was 73, 35, 8, 11, 5, and 7% of 74 RCTs respectively; the items in STRICTA of the numbers of needles inserted, the needle type, the depths of insertion, the length of clinical experience, and the background of the acupuncture practitioners was 5, 47, 35, 1, and 8% of 74 reports, respectively. Compared with zhang’s study [9], the quality of reporting RCTs of EA for stroke in present study is better. In 2014, Zhuang et al. [10] analyzed the quality of reporting of only 15 RCTs on acupuncture for subacute and chronic stroke, indicating that poor reporting existed in terms of outcomes, sample size, outcomes and estimation, ancillary analyses, with positive rate less than 30% according to CONSORT statement. Meanwhile, based on STRICTA statement, item 4a: Details of other interventions and 4b: Setting and context of treatment, the positive rate was 20 and 33% respectively. The quality of reporting of RCTs on EA for stroke in present study is similar to the results of Zhuang’s study [10]. This result indicates some improvements in the quality of reporting of RCTs on both acupuncture and EA for stroke. One probable reason is that reporting of several important aspects of trial methods improved because the endorsement of the CONSORT Statement and STRICTA statement. Another possible reason is that Zhuang [10] studied only a small number of selected RCTs, thus the conclusions may not be scientifically sound and may be misleading. For present EA study, the third possible reason is that EA is more readily controlled, standardized and objectively measurable. Additionally, EA is mainly considered as a method to provide stronger treatment for nervous and mental diseases like stroke. Thus, the use of EA for stroke research can at least in part improve the standards of published RCTs and is favored in stroke trials.

From the comparison of the included studies published in Chinese and in English, we found the compliance with CONSORT statement is unsatisfactory. Thus, reporting of RCTs both in English and in Chinese should endorse the CONSORT items as complete as possible. In particular, studies published in Chinese need to improve the reporting of (1a) title, (11a) blinding, and (13b) losses and exclusions. For the STRICTA statement, the proportions of fulfilling the items (1b) reasoning for treatment and (2d) response sought in Chinese have statistically significant increase compared with those in English. The main reasons are as follows: (1) acupuncture has been practiced in China for over 2000 years [89] and Chinese journals lay emphasis on reasoning for treatment; (2) as one of the fundamental characteristics of acupuncture, *deqi* has been used as a prerequisite for clinical effects for a long time in China [90]. However, the proportion of reporting item (5) practitioner background achieved statistically significant improvement in English compared that in Chinese. The possible reason is that English journals pay more attention to endorsing the STRICTA statement [91]. Thus, both English and Chinese journals need to endorse reporting acupuncture RCTs based on the STRICTA checklist, especially item (5) practitioner background in Chinese and items (1b) reasoning for treatment and (2d) response sought in English, thereby actualizing an improvement in reporting quality of RCTs for acupuncture.

There are some limitations in this study. First, the searching languages are limited to only Chinese and English during sample selection. The reports which are published in other languages may be left out, and may harm the reliability of our results. Second, we only discussed the reporting quality of RCTs on EA in the present study, and compared with that of RCTs on acupuncture in the previous studies. The results may be potentially misleading, and the direct comparison between the reporting qualities of RCTs on manual acupuncture for stroke with that of RCTs on EA is needed in the future. Third, we carried out data extraction based on the published paper itself. This approach meant that we were unable to capture some primary trials with truly good quality in trial methodology but poor reporting in the final publication. Thus, when assessing trial quality of such studies, reviewing research protocols and contacting trialists for more information are needed.

## Conclusions

Our study indicated that the overall quality of reporting of RCTs on EA for stroke according to CONSORT and STRICTA statement was moderate and the reporting quality needs further improvement. In particular, it must be emphasized that the poor quality reporting of crucial items which includes sample size calculation, sequence generation, allocation concealment, randomization implementation, blinding, and practitioner background should be adequately involved in RCTs on EA for stroke. More attention should be given to the reporting of RCTs on EA for stroke to ensure that all items in checklist of CONSORT and STRICTA are clearly delineated, especially the central items in the methodology. In addition, the use of EA for stroke research can possibly improve the standards of published RCTs when compared with manual acupuncture trials. However, this need further direct comparative studies.

## Abbreviations

CAM: Complementary and alternative medicine
CBM: Chinese Biomedical Database
CI: Confidence interval
CNKI: China National Knowledge Infrastructure
CONSORT: Consolidated standards of reporting trials
EA: Electroacupuncture
ICH: Intracerebral hemorrhage
ITT: Intention-to-treat
RCTs: Randomized controlled trials
STRICTA: Standards for reporting interventions in clinical trials of acupuncture.
